# Review for the generalist: evaluation of low back pain in children and adolescents

**DOI:** 10.1186/1546-0096-8-28

**Published:** 2010-11-22

**Authors:** Kristin M Houghton

**Affiliations:** 1 K4-123 ACB BC Children's Hospital, 4480 Oak Street, Vancouver, Canada, V6H 3V4

## Abstract

Back pain is common in children and adolescents. Most cases of back pain are non-specific and self-limiting. In children and adolescents, pain is usually related to the posterior elements of the spine and disc-related problems are rare. Serious pathology, including malignancy and infection needs to be excluded. Evaluation and management is challenging and requires a thorough history and physical exam, and understanding of the immature skeleton. Diagnostic imaging is useful in the evaluation of a child or adolescent with low back pain and can help guide management. This article will review common causes of back pain in the pediatric population.

## Background

Back pain is common in young people with one year prevalence rates varying from 7% to 58% [1]. The lifetime prevalence may be as high as 70-80% by age 20[2]. Low back pain (LBP) in childhood/adolescence is a significant risk factor for LBP in adulthood[3].

Low back pain (LBP) is more common in girls, with increasing age, with high and low levels of activity, and during periods of rapid growth. LBP is particularly common in youth participating in sports requiring flexion/extension/rotation of the spine (gymnastics, wrestling, football, racquet sports, diving, volleyball, cricket)[4]. Familial LBP, psychological distress and behavioural factors are also associated with LBP[5]. Greater relative backpack weight is associated with upper- and mid-back pain but not LBP. Research supports the use of a 10% of body weight cutoff for safe use of backpacks [6].

The etiology of back pain in children and adolescents differs significantly from the adult population. Pediatric dogma suggests that children with back pain may have serious pathology, including malignancy and infection [7]. However, most cases of back pain are non-specific and self-limiting. A recent prospective study of 73 children under age 18 years, with back pain of greater than 3 months duration found only 21% of the patients had positive findings after diagnostic evaluation or a minimum of 2 years follow-up. Spondylolysis with or without spondylolisthesis was the most common diagnosis [8]. Back pain in young athletes is usually related to the posterior elements of the spine and disc-related problems are rare [9]. Evaluation and management is challenging and requires a thorough history and physical exam, understanding of the pediatric skeleton and appropriate diagnostic imaging. This article will review common causes of low back pain in the pediatric population. It is not meant to be an exhaustive review and will not review acute trauma.

## Clinical history

Children and adolescents frequently present with diffuse, poorly localized lumbar pain in the absence of associated neurologic symptoms. Focal pain and neurologic symptoms are more likely to represent underlying pathology. Onset of symptoms will help differentiate between acute trauma and chronic overuse injury, postural and developmental abnormalities. The clinical history should include a thorough description of the pain characteristics (location, character, onset, duration, change with activity or rest, aggravating and alleviating factors, night pain); trauma (acute macrotrauma, repetitive microtrauma, athletic activities, recent/remote); mechanical symptoms (worse during or after activity); inflammatory symptoms (morning stiffness, better with movement); systemic symptoms (fever, night sweats, weight loss); neurological symptoms (radiculopathy, weakness, bowel or bladder dysfunction); gait (foot drop); effects of previous treatments and the current level of function of the child. Lifestyle, psychosocial factors, interference with school, backpack weight, and family history of back pain should also be considered.

A history of previous injury or surgery, treatment with immunosuppressive agents, scoliosis, osteoporosis, malignancy, neurological disorder, or chronic inflammatory joint disease is significant. Family history of orthopedic, neurologic, rheumatic and HLA-B27 associated diseases (ankylosing spondylitis, reactive arthritis, psoriasis, inflammatory bowel disease) is also important.

## Anatomy and physiology

Back pain can be localized to the low back (lumbar spine), mid back (thoracic spine) or neck (cervical spine). The main functions of the spine are to protect the spinal cord and nerve roots, support and balance the body, and allow flexibility and mobility. Movement of the axial spine occurs in three planes: flexion/extension, lateral flexion, and lateral rotation. The SI joint is a diarthrodial joint with hyaline cartilage on the sacral side and fibrocartilage on the iliac side. It also contains numerous ridges and depressions, indicative of its function for stability more than motion. Limited motion of the SI joints occurs with the two sacroiliac joints moving together as a single unit.

## Physical examination

During the clinical assessment the physician should try and reproduce the patient's pain through palpation and movement. Examination of posture, stance, core strength and hamstring flexibility is important in determining any potential predisposing or contributing factors. A complete neurological exam should include trunk and extremity strength, pain, proprioception and deep tendon reflexes. Examination of the hips, abdomen and pelvis is important to rule out referred pain.

### 1. Observation

#### Standing

Posture, pelvis heights, lower limb alignment, foot arch (cavus, planus), midline skin markings are observed with the patient standing. The standing posture of the spine is mild cervical lordosis, thoracic kyphosis and lumbar lordosis. Both anterior superior iliac spines (ASIS) and posterior superior iliac spines (just below the dimples of Venus) should be in the same horizontal plane; if they are not there may be pelvic obliquity. An exaggerated lumbar lordosis may be due to weak abdominal muscles or hip flexion contracture.

#### Supine or sitting

Lower limb lengths and alignment can be assessed in supine or sitting position.

### 2. Range of motion

Active movements of the thoracolumbar spine are tested in standing position with the pelvis/iliac crest stabilized. Flexion - patient bends as far forward as possible with knees straight. (measure distance from fingertips to floor if patient can not touch the floor).

Extension - patient bends as far backward as possible with knees straight and lumbar spine supported by examiner. (30 degrees) Lateral flexion - patient bends to the side as far as possible. (should be able to touch fibular head) Lateral rotation - in addition to stabilizing the pelvis with one hand on the iliac crest, the examiner may place a hand on the opposite shoulder. The patient rotates the trunk as far as possible. (30 degrees).

### 3. Palpation

Surface anatomy of the posterior aspect of the spine is best appreciated with the patient standing. It is important to palpate specific structures. The point of maximal tenderness should be correlated with the underlying bone or soft tissue anatomy. Palpate over the spinous processes, facet joints, paraspinal muscles, sacroiliac joints, gluteal muscles, posterior superior iliac spines, posterior iliac crest, ischial tuberosities and greater trochanters.

Surface anatomy of the anterior aspect of the spine is best appreciated with the patient supine with the knees bent to relax the abdominal muscles. The vertebral bodies of L4, L5 and S1 are palpable just below the umbilicus. The anterior abdominal muscles are best palpated with the patient performing a partial sit-up.

### 4. Special tests

#### Scoliosis test (Adams Forward Bend Test)

Patient standing and instructed to forward flex with the feet together and knees straight. The curve of structural scoliosis is more apparent when bending over and the examiner may observe an imbalanced rib cage, with one side being higher than the other.

#### Modified Schober's test for lumbosacral spine mobility

**Figure 1:**
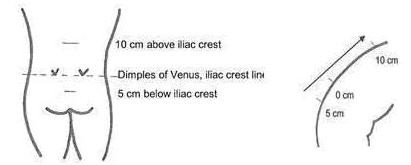
**Modified Schober's Test**. Patient standing and measurements made 10 cm above and 5 cm below the lumbosacral junction (dimples of Venus, iliac crest line). Repeat measurement with patient in full forward flexion.

Patient standing and measurements made 10 cm above and 5 cm below the lumbosacral junction (dimples of Venus). Repeat measurement with patient in full forward flexion. In general, the measure should increase by at least 6 cm to 21 cm[10]. An increase of less than 6 cm suggests decreased lumbar spinal mobility, which may be seen in spodyloarthropathies. (Figure 1)

#### Straight leg raise

Patient supine and the examiner lifts the leg keeping the knee straight, while supporting the calcaneus. Normal range is 80 degrees. If the patient has tight hamstrings, they may localize pain to the hamstring area. If the patient has sciatic pain, the patient may experience pain extending down the back of the leg or in the back. Lowering the leg 15 degrees and dorsiflexing the foot to stretch the sciatic nerve should reproduce sciatic pain. If pain persists it is sciatica, if not it is likely tight hamstrings.

#### FABER or Patrick test

Patient lying supine and leg passively brought into Flexion, ABduction, External Rotation with foot resting on opposite knee. Press down gently but firmly on the flexed knee and the opposite anterior superior iliac crest. Pain may be felt in the groin with intraarticular hip pathology or SI joint region with SI pathology.

#### Trendelenburg Test

Positive when patient stands on one leg and the contralateral hip drops, indicative of gluteals/hip abductor weakness. There are many conditions that may weaken the hip abductors including musculoskeletal and neurologic problems.

### 5. *Flexibility*

The back and hips should be moved through active and passive range of motion.

Hamstring flexibility is determined by measuring the popliteal angle. The hips and knees are flexed to 90 degrees and then the examiner extends the knee until there is firm resistance. An angle greater than 45 degrees or asymmetry is common with tight hamstrings and low back pain.

### 6. *Strength*

Lower extremity strength and core strength should be examined.

### 7. Peripheral joint exam

Range of motion. Further detailed exam as appropriate.

### 8. Neurological exam

Deep tendon reflex tests [Patellar (L2, 3, 4), Achilles (S1)].

Superficial reflexes [Abdominal reflex (T7-T10 and T10 -L1for upper and lower muscles)]

Upper motor neuron reflexes [Cremasteric reflex (T12), Anal wink (S 2, 3, 4)]

Pathologic reflexes [Babinski test]

Sensation of the lower leg in major dermatomes.

Pain and proprioception.

### 9. Abdominal and pelvic exam [abdomen and pelvis pathology may refer pain to the back]

## Investigations

Laboratory tests are necessary in evaluating patients with back pain and a high suspicion of infection or systemic disease. CBC, ESR or CRP, blood and joint cultures should be done if infection is suspected. Arthritis is a clinical diagnosis; ANA, rheumatoid factor and HLA-B27 are helpful in classification and treatment but not diagnosis. CBC, peripheral smear should be done if hematological malignancy is suspected.

## Imaging

### Radiographs

Standard thoracolumbar views include the standing anteroposterior (AP) and lateral projections.

### Technetium bone scan

Bone scan identifies areas of increased osteoblastic activity and can help localize subtle areas of bone injury that may not be visible on radiographs. SPECT (single-photon emission computed tomography) is recommended for imaging the spine in patients who have negative radiographs and no neurological findings. SPECT is especially useful in identifying stress fractures and spondylolysis [8].

### Computed (CT)

CT provides additional bony and cartilage detail. CT is useful in further characterizing lesions identified on bone scan, including fractures, spondylolysis and tumors [11].

### Magnetic resonance imaging (MRI)

MRI provides increased soft tissue contrast allowing evaluation of the spinal cord and paraspinal structures. MRI is useful in the diagnosis of back pain with neurologic findings but anatomic localization on clinical exam is necessary to increase the specificity of imaging.

## Pain from the posterior elements of the spine

### Spondylolysis and Spondylolithesis

Spondylolysis is a defect in the pars interarticularis and most commonly affects the fifth and fourth lumbar vertebrae. The majority of pediatric cases represent a fatigue or stress fracture in the pars interarticularis. The incidence of pars defects on plain radiographs is approximately 6% of the general population by age 18 and 8% to 15% of elite adolescent athletes [12]. Spondylolysis is more common in boys and in athletes participating in sports involving repetitive extension, flexion, and rotation. Acute and overuse injuries both occur, with overuse injuries being more common. There is also a genetic predisposition to spondylolysis among certain ethnic groups with the prevalence among the Inuit in Northern Canada as high as 20-50% [13]. An association between spina bifida occulta and spondylolysis has also been reported [13].

Spondylolithesis occurs with bilateral pars defects and is defined by forward translation of one vertebra on the next caudal segment. Spondylolithesis is graded based on the percentage of slip of one vertebral body on the vertebral body below [grade 1 slip (0-25% slip), grade 2 (25-50%), grade 3 (50-75%) and grade 4 (> 75%)] [14]. Spondylolithesis is more common in females, and typically occurs during the adolescent growth spurt. Progression of slip after skeletal maturity is rare [14].

Older children and adolescents usually present with the insidious onset of low back pain, worsened by extension, activity or prolonged standing. Radicular symptoms and neurologic deficit affecting the L5 or S1 nerve roots may be present with nerve root irritation and high grade spondylolisthesis. On examination, patients may have an exaggerated lumbar hyperlordosis, ipsilateral paraspinal muscle spasm and tight hamstrings. Spinal extension is typically painful, especially single leg extension on the affected side. There is often limited forward flexion and straight leg raise. There may be focal tenderness to palpation over the site of the pars lesion and a step-off at the lumbosacral junction with spondylolisthesis.

**Figure 2:**
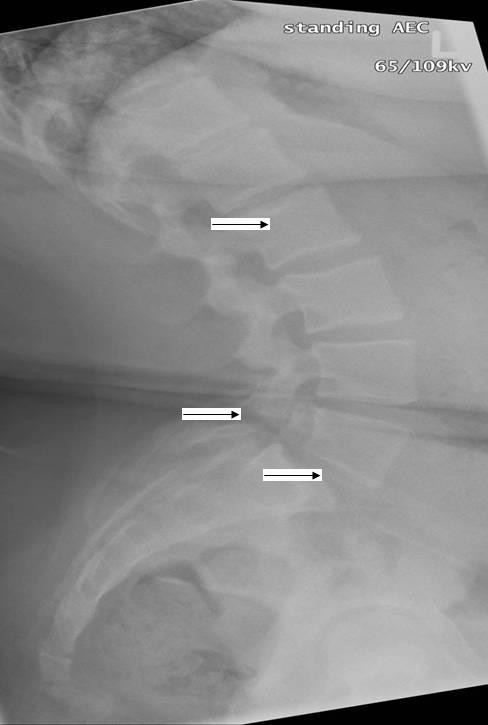
**Lateral radiograph of the lumbosacral spine in a 12 year old obese girl with low back pain**. Bilateral pars defects (middle arrow) are present and there is Grade I spondylolisthesis (bottom arrow) of L5 on S1. There is also evidence of Schmorl's nodes (top arrow) within the lower thoracic spine. (Radiograph courtesy of BC Children's Hospital).

Investigations include radiographs (standing lateral, AP) to characterize gross bony abnormalities and stage spondylolisthesis. (Figure 2) Single-photon emission computed tomography (SPECT) bone scan is more sensitive and identifies pars lesions with active bone turnover. CT scans are performed thru the abnormal area identified on SPECT to confirm lesions and acuity. The role of MRI is unclear with some studies citing a high rate of false positives and others showing good correlation with SPECT and CT [15].

Management includes activity restriction until asymptomatic and physiotherapy focusing on core strength and hamstring flexibility. The role of braces is controversial with studies showing bony healing with the use of a rigid brace, a soft brace, or no brace [15].

The majority of adolescents return to activities by 6 months after diagnosis. Skeletally immature children and adolescents with bilateral spondylolysis or spondylolisthesis require standing lateral radiographs to assess for slip progression every 6 to 12 months until skeletal maturity is reached. Adolescents with greater than 50% slip (Grade 3 or 4) warrant orthopedic referral and possible surgical treatment.

### Posterior element overuse syndrome

If spondylolysis has been ruled out, posterior element overuse syndrome should be considered. Posterior element overuse syndromes include injury to the muscle-tendon units, ligaments, joint capsules and facet joints. It is also referred to as spondylogenic back pain, hyperlordotic back pain, mechanical low back pain and lumbar facet syndrome [9]. Children and adolescents present with extension related low back pain and often have weak core muscles, tight hamstrings and limited lumbar range of motion on clinical exam. Investigations are negative for spondylolysis. Management includes ice and non-steroidal anti-inflammatory drugs, modified activity and physiotherapy focusing on core strength and hamstring flexibility.

## Sacroiliac joint pain

The sacroiliac (SI) joint has limited motion but can be a source of pain if there is excessive or reduced motion. Altered mechanics of the lumbar spine may cause muscle spasm and resultant stress to the SI joints [9]. Inflammation (see inflammatory disorders) or stress fracture of the sacrum may also cause SI joint pain. Children and adolescents present with the insidious onset of low back and buttock pain, worsened by extension, prolonged standing or sitting. On clinical examination there may be pain to palpation over the SI joint and pain with lumbar extension and FABER test. Mobility of the SI joint may be increased or decreased. There may be weakness of the gluteal muscles and positive Trendelenberg test. Radiographs of the pelvis are often normal. Bone scan may detect stress fracture and the presence of bone marrow edema on MRI will better define the anatomic site of abnormality. Management includes ice, non-steroidal anti-inflammatory drugs, activity modification, bracing and physiotherapy.

## Pain from the anterior elements of the spine

### Scheuermann's Disease

Scheuermann's disease is the most common cause of kyphotic deformity in adolescence with a reported incidence of 1-8% [16]. It affects male and females in equal numbers and the etiology is unknown [16]. The onset usually occurs just prior to puberty and is commonly attributed to poor posture, causing delay in diagnosis and treatment. Most adolescents are asymptomatic and cosmetic appearance is their chief presenting complaint. However, pain may be present, especially with prolonged sitting posture and exercise. On clinical examination, the kyphosis may be thoracic or thoracolumbar. The kyphosis is fixed and remains evident on hyperextension and flexion of the spine, distinguishing Scheuermann's from postural kyphosis which disappears with forward flexion. The thoracic pattern is most common; adolescents have non-structural hyperlordosis of the lumbar and cervical spine and may have moderate scoliosis. The thoracolumbar form is uncommon and is associated with relative lumbar hypolordosis and pain. Tightness of the anterior shoulder muscles, iliopsoas and hamstrings is common [17]. Neurologic deficits are rare and cardiopulmonary insufficiency occurs only with severe curves measuring > 100 degrees [18]. Standing lateral and anterior-posterior radiographic features classically show anterior wedging of at least three adjacent vertebral bodies by 5 or more degrees, end plate irregularities, loss of disc space height, Schmorl nodes and Cobb angle of at least 45 degrees [19]. (Figure 2)

Observation and symptomatic treatment with extension exercises, hamstring and pectoral muscles is sufficient for minor curves. Bracing is indicated for thoracic curves greater than 55 degrees and thoracolumbar curves greater than 40 degrees. Surgical intervention may be considered for adolescents with curvature of 70 degrees or greater, refractory pain or neurologic deficit [20].

### Lumbar Disk Herniation

Lumbar disc herniation in pediatric and adolescent populations is uncommon with an incidence of 3.5% recently reported [21]. Disk protrusion (primarily L4-5 or L5-S1) is more common in adolescents than in young children. In a study of 100 consecutive adolescent athletes presenting with acute low back pain, only 11% had disc pathology[22]. Children and adolescents with disc herniation present with low back, buttock or hip pain worsened by bending forward, coughing, sneezing or straining with bowel movements. On examination, there is often limitation of forward flexion and positive straight-leg raise test. Weakness in a myotomal distribution, numbness in a dermatomal distribution and diminished reflexes are not common in adolescents with disc herniation. Investigations include radiographs and MRI of the lumbar spine [11]. Careful inspection is required to differentiate between spondylolysis with nerve root impingement or disc herniation with nerve root impingement. Symptomatic treatment with rest, analgesics, and physical therapy is successful in the majority of patients but adolescents do not fare as well with conservative therapy as do adults. Early microdiscectomy for patients who do not respond to conservative treatment is advocated by some surgeons while others reserve operative therapy for progressive neurologic deficit [23].

## Scoliosis

Idiopathic scoliosis affects 1-3% percent of children and adolescents [24]. On clinical examination, Adams Forward Bend Test will elicit the curve of structural scoliosis and measurement of a Cobb angle of at least 10 degrees on a standing coronal radiograph confirms the diagnosis. Scoliosis is usually painless; however one study reported back pain in up to 23% at presentation [25]. Untreated scoliosis may also cause back pain [24]. Management is determined on an individual patient basis and is dependent on skeletal maturity and degree of the curve. Expectant observation, physiotherapy, orthoses and surgery are potential management strategies.

## Infectious disorders

### Discitis and vertebral osteomyelitis

Childhood discitis is uncommon and usually affects the lumbar region in children younger than 5 years [26]. The etiology is thought to be a bacterial infection, usually Staphylococcus aureus [27]. Children may present with irritability, refusal to bear weight, a limp, back pain or abdominal pain. Fever is not always present. On clinical examination, toddlers may refuse to stand or sit. Children have pain on flexion or refusal to flex the spine. There may be loss of lumbar lordosis. There is seldom point tenderness to palpation of the spine. Complete blood count and inflammatory markers are normal or mildly elevated and blood cultures are negative which may lead to a delayed diagnosis. Radiographs demonstrate disc space narrowing and irregular endplates of adjacent vertebrae but these changes are not evident until 2 - 4 weeks. Technetium bone scan shows focal uptake earlier. MRI is the diagnostic imaging of choice as it detects discitis and can exclude paraspinal and spinal tumors [11]. Most children have rapid improvement with a 4 to 6 week course of antibiotic and only children refractory to antibiotics should undergo biopsy of the infected disk space. Although, the natural history is benign in most children, plain radiographs should be done at regular intervals for 12 to 18 months to ensure the destructive process resolves [27].

Vertebral osteomyelitis is uncommon in children, representing only 1-2% of all cases of pediatric osteomyelitis. School age children and adolescents are most commonly affected. Children and adolescents usually present with back pain, muscle spasm and fever. Complete blood count may show elevated white blood cell count and inflammatory markers may be high. Blood culture and/or biopsy may be positive for a pathogen, with Staphylococcus aureus most common. Tuberculosis should be considered in endemic areas. Radiographs demonstrate bony destruction but these changes may not be evident for 2 - 4 weeks. MRI is the diagnostic imaging of choice with high sensitivity and specificity [11]. Management includes a 4 to 6 week course of antibiotic, rest and/or immobilization and possible surgical debridement.

## Inflammatory disorders

Spondyloarthropathy includes a group of disorders with arthritis and enthesitis affecting the spine and sacroiliac joints. Spodyloarthropathies are more common in boys and are frequently associated with the genetic marker HLA-B27. Spine and sacroiliac joint involvement is more common in the juvenile idiopathic arthritis subtype Enthesitis Related Arthritis (ERA) than other childhood arthritides, but axial disease is often a late manifestation. Reactive arthritis, psoriatic arthritis and inflammatory bowel disease may also present with spondyloarthropathy. Children may present with back or buttock pain but the typical history of morning stiffness, gradual resolution of pain with activity and clinical exam findings of limited lumbar mobility (Schober test), sacroiliac joint tenderness and peripheral enthesitis or arthritis usually allow the practitioner to make the correct diagnosis [10]. A complete joint and systemic examination to exclude other joint involvement is important. Radiographs of the lumbar spine and SI joints are often normal early in disease but widening of the joint, sclerosis and erosions may be apparent. MRI with gadolinium may delineate early inflammatory change.

Treatment of arthritis includes physiotherapy for improving range of motion and strength, NSAIDs, disease modifying anti-rheumatic therapy, or biologic therapy. All children and adolescents suspected of having inflammatory arthritis should be referred to a pediatric rheumatologist.

## Pain amplification syndromes

Many pediatric patients with severe chronic musculoskeletal pain do not have an identified cause. The cause of amplified musculoskeletal pain is unknown, but minor trauma, underlying chronic illnesses and psychological distress have been associated. Typically, an affected child has pain at multiple sites in addition to back pain.

On physical examination, children may have altered posture, manifest allodynia (pain generated by normally non-painful stimuli), and have tenderness at multiple bony and soft tissue sites. Examination maneuvers that may help differentiate organic from non-organic pain include simulation of axial loading and passive rotation, and distracted straight leg raising [28]. General and neurovascular exam are normal. Diagnostic studies are typically not helpful, other than to rule out other pathology. Treatment includes aggressive physiotherapy for desensitization, range of motion and function and multidisciplinary team approach to help with pain coping mechanisms.

## Tumors

Benign and malignant tumors are rare causes of back pain. Children may complain of severe pain and night pain and there may be associated fever, weight loss, neurologic symptoms or signs. Symptomatic benign bone lesions include osteoid osteoma, osteoblastoma, Langerhans Cell Histiocytosis, bone cysts and non bacterial chronic osteomyelitis. Malignant bone tumors include local Ewing's sarcoma, osteosarcoma, leukemia or metastases from neuroblastoma or rhabdomyosarcoma. Chondrosarcoma and synovial sarcoma may also present with back pain. Imaging and laboratory tests will help confirm the diagnosis.

## Conclusion

Back pain is common in the pediatric population. Most cases of low back pain are non-specific and self-limiting but serious pathology including infection and malignancy need to be considered. Pain is usually related to the posterior elements of the spine and disc-related problems are rare. Diagnostic imaging is useful in the evaluation of a child or adolescent with low back pain and can help guide management.

## Competing interests

The author declares that they have no competing interests.

## Author's information

KH is certified in both pediatric rheumatology and sports medicine and has a Masters of Science in Human Kinetics.
